# Challenges, Threats, Security Issues and New Trends of Underwater Wireless Sensor Networks

**DOI:** 10.3390/s18113907

**Published:** 2018-11-13

**Authors:** Guang Yang, Lie Dai, Zhiqiang Wei

**Affiliations:** 1 School of Information Science and Electrical Engineering, Shandong Jiaotong University, Jinan 250357, China; dailie@sdjtu.edu.cn; 2 College of Information Science and Engineering, Ocean University of China, Qingdao 266100, China; weizhiqiang@ouc.edu.cn

**Keywords:** underwater communication, underwater wireless sensor networks, security, UWSNs

## Abstract

With the advances in technology, there has been an increasing interest from researchers and industrial institutions in the use of underwater wireless sensor networks (UWSNs). Constrained by the open acoustic channel, harsh underwater environment, and their own particularities, UWSNs are vulnerable to a wide class of security threats and malicious attacks. However, most existing research into UWSNs has not taken security into consideration. Moreover, the existing relatively mature security mechanisms for WSNs cannot be directly utilized in UWSNs. For these reasons, this article aims to present a comprehensive overview of the particularities, constraints, attacks, challenges and current security mechanisms of UWSNs. In addition, challenging, open and hot research topics are outlined.

## 1. Introduction

Underwater wireless sensor networks (UWSNs) have proven strength in various underwater applications of ocean monitoring, resource exploration, surveillance and military use in harsh underwater environments [1,2].

**Figure 1:**
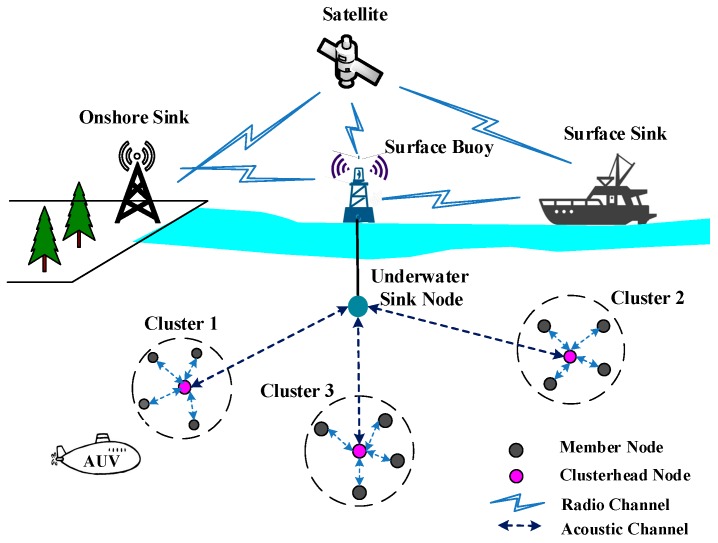
Underwater wireless sensor network (UWSN) architecture.

As shown in Figure 1, UWSNs are composed of several components: onshore sink, surface buoy, underwater sink node, and underwater sensor nodes. Moreover, satellite, vessel, and autonomous underwater vehicles (AUVs) can be used to expand the sense and communication range. Underwater sensor nodes monitor physical or environmental conditions, such as pressure, sound, temperature, etc. and cooperatively transmit data to the underwater sink node. The data are transmitted to a surface buoy via wired link, and finally received at an onshore sink or surface sink via radio communication. There are three different architectures for UWSNs. Static two-dimensional architecture: all the nodes are anchored to the ocean floor. The underwater sink node collects data from sensor nodes by the horizontal transceiver. Then, it relays data to surface buoy by the vertical transceiver or wired link. Static three-dimensional architecture: underwater nodes are anchored to the seabed and fitted out with floating buoys. The buoy pays the sensor towards the water surface. The lengths of the cables are different for the required depth of sensor nodes. Three-dimensional architecture with AUVs: as discussed above, AUVs can be used to expand the sense and communication range. The AUVs could be considered as super nodes, which have more energy, can move independently, and could be routers between fixed sensors, managers for network reconfiguration, or even a normal sensor.

In UWSNs, to prolong the lifetime of whole network, cluster-based network architecture is widely used. A cluster-head (CH) node is elected to be the sink-node of the cluster, which aggregates and relays packets intra-cluster and inter-cluster. Hence, the energy consumption of CH is greater than member nodes. Sensor nodes are elected to be the CH in turn to balance the energy consumption and further prolong the lifetime of the whole network. The election protocol may be based on the residual energy [3], the best energy consumption [4], or the optimal number of CHs [5].

Existing research on UWSNs is mainly focused on communication, self-organization, processing capabilities, cover ability, connectivity, adaptability and low energy consumption. Unfortunately, this existing research is constrained in terms of countering security threats in UWSNs because the resources are much more constrained while the security situation is more server-based due to the particularities and networking environments [6].

The contributions of this article are as follows:

- In this article, the special particularities and constraints of UWSNs and underwater acoustic channels are presented and discussed in detail. Based on the analyses, we conclude that UWSNs are vulnerable to various threats and attacks and security issues should be discussed.
- Threats and attacks in UWSNs are classified and discussed in this article. In addition, denial of service (DoS) attacks and feasible countermeasures in each layer are analyzed in detail.
- Compared with WSNs, some especial security requirements of UWSNs are discussed and existing security mechanisms and specific protocols are presented.

The remainder of this paper is organized as follows. In Section 2, the peculiarities of UWSNs and the underwater acoustic network environment are introduced. The threats and challenges in UWSNs are discussed in Section 3. The security requirements and current security researches for UWSNs are presented in Section 4. In Section 5, open research problems and future research topics are outlined. Finally, Section 6 concludes the paper.

## 2. Particularities and Constraints

As a branch of wireless sensor networks (WSNs), some particularities of UWSNs are similar to WSNs [7,8]. Unfortunately, due to the harsh working environment, there are some special particularities and constraints, which are outlined below.

### 2.1. Extremely Limited Resources

Underwater sensor nodes are extremely limited in hardware resources, including energy, computational capability and storage space. Due to higher distances and more complex signal processing at the receivers to compensate for the attenuation of the signal, the power consumed for underwater acoustic communication is much higher than in terrestrial radio communication. Underwater sensor nodes are deployed in shallow or deep water, where it is inconvenient to charge or replace the nodes’ battery. In the case of saving energy consumption to prolong the network lifetime, the computational capability and storage space are constricted by the energy consumption problem. Hence, virtually all existing research and technologies for UWSNs focus on saving energy consumption at the expense of capability and security.

### 2.2. Unreliable Communication Channel

On account of the nature of the transmission medium and physical properties of the environments, underwater acoustic channel is temporally and spatially variable.

#### 2.2.1. Long and Variable Propagation Delay

The propagation speed of underwater acoustic wave is approximately 1.5 × 10^3^ m/s, which is five orders of magnitude lower than the radio propagation speed (3 × 10^8^ m/s) in air. Moreover, the speed is affected by some factors including the temperature, depth, and salinity, which can be calculated by the equations below [9]:

(1) $$V_{1}=1449.2+4.6T+0.055T^{2}+0.00209T^{3}+\left(1.34-0.01T\right)\left(S-35\right)+0.06D$$

(2) $$V_{2}=1449+4.6T+0.055T^{2}+0.003T^{3}+\left(1.39-0.012T\right)\left(S-35\right)+0.017D$$

(3) $$V_{3}=1449.2+4.6T-0.055T^{2}+0.00029T^{3}+\left(1.34-0.01T\right)\left(S-35\right)+0.016D$$

(4) $$V_{4}=1448.96+4.591T-0.05304T^{2}+0.0002374T^{3}+\left(1.34-0.0102T\right)\left(S-35\right)\;+0.0163D+1.675\times 10^{-7}D^{2}-7.139\times 10^{-13}TD^{3}$$

(5) $$V_{5}=1492.9+3\left(T-10\right)4.6T-0.006\left(T-10^{-2}\right)-0.04{\left(T-18\right)}^{2}\;+\left(S-35\right)\left(1.39-0.01T\right)+D/61$$

where *V* is acoustic speed in m/s, *T* is temperature in degrees Celsius, *S* is salinity in parts per thousand, and *D* is depth in meter. From the above, using different equations will get different acoustic speed. To simplify analysis and calculation, it is often assumed that the salinity and temperature is constant. Unfortunately, in coastal areas or near rivers, these assumptions are generally invalid making make the acoustic propagation speed variable. Moreover, the propagation delay is long and variable, and can be calculated as follows:

(6) $$t=\frac{d}{v}$$

where *t* is propagation delay in second, *d* is distance between sender and receiver in meter, and *v* is acoustic speed in m/s. As discussed above, due to a long propagation delay and variable sound speed, the time synchronization and the localization are difficult to achieve in the underwater scenario.

#### 2.2.2. Limited Bandwidth and Low Data Rates

The available bandwidth of underwater acoustic channels is limited and dramatically depends on both transmission range and depth.

As shown in Table 1, for long range communication in deep water, the available bandwidth ranges from 500 Hz to 10 kHz; for medium range communication in shallow water, the available bandwidth ranges from 10 to 100 kHz; and for short range communication in deep water, the available bandwidth ranges from 100 to 500 kHz. The available bandwidth becomes much wider with the decrease of communication range, especially at ranges less than 100 m.

As shown in Table 1, the data rate is also relevant with communication range. For long range communication, the maximum data rate is approximately 10 kb/s. For medium range communication, the maximum data rate is approximately 50 kb/s. For short range communication, the maximum data rate can reach more than 100 kb/s. There is a tradeoff between data rate and channel bandwidth. Because underwater acoustic communication is possible only over limited bandwidths, the data rate of the underwater acoustic channel is much slower than radio channel in the air. In many UWSN applications including AUV control, a larger communication range is more important than a higher transfer rate.

#### 2.2.3. Ambient Noise

Ambient noise is another factor that severely influences the communication in underwater acoustic channel. The ambient noises include: the radiated noises and self-noises from vessels, noises caused by waves and other surface motions; turbulence noise, thermal noise, and the noises that come from marine animals. These ambient noises can be classified into four types that affect acoustic communication at different frequency band, including turbulence noise (*N_t_*, less than 10 Hz), shipping noise (*N_s_*, 10–100 Hz), wind noise (*N_w_*, 100 Hz–100 kHz), and thermal noise (*N_th_*, over 100 kHz) [10]. These ambient noises can be calculated by the following empirical equations in dB, which is a function of frequency:

(7) $$10\log N_{t}\left(f\right)=17-30\log f$$

(8) $$10\log N_{s}\left(f\right)=40+20\left(S-0.5\right)+26\log f-60\log\left(f+0.03\right)$$

(9) $$10\log N_{w}\left(f\right)=50+7.5w^{\frac{1}{2}}+20\log f-40\log\left(f+0.4\right)$$

(10) $$10\log N_{th}\left(f\right)=-15+20\log f$$

where *w* is the speed of sea-surface wind in m/s. Based on these four ambient noises, the total noise can be calculated with the following equation:

(11) $$N\left(f\right)=N_{t}\left(f\right)+N_{s}\left(f\right)+N_{w}\left(f\right)+N_{th}\left(f\right)$$

The ambient noises may cause critical effects upon sonar performance and large fluctuations upon a change in time, location or depth, which may influence the availability of the network.

### 2.3. Transmission Loss

During spreading, the energy of acoustic signal may be attenuated and absorbed by the medium. The transmission loss includes spreading loss and attenuation loss.

Spreading loss (SL): this is the power loss during the spreading period from source node to destination node. In the spreading period, the acoustic wave front will occupy a larger and larger surface area, and therefore the wave energy in each unit surface becomes less and less. According to the source and working environment, the spreading power loss can be modeled by two methods, including spherical spreading and cylindrical spreading. As shown in Table 2, the acoustic wave loss model includes spherical wave loss model and cylindrical wave loss model.

Spreading loss can be calculated with the following formula:

(12) SL=d×10logr

where *d* is the spreading factor that describes the loss model, and r is the range in meter.

As shown in Table 3, the factor d is commonly set to 2 for spherical wave loss model, and set to 1 for the spherical wave loss model. But for a practical underwater application, the spreading loss is a hybrid of spherical and cylindrical spreading, where d is set to 1.5 [11].

Attenuation loss (AL): during the propagation period, the energy of an acoustic wave would be converted to other forms (e.g., heat) and absorbed by the transmission medium. Moreover, the attenuation loss is dependent on frequency. Hence, the absorption coefficient *a*(*f*) can be used to express and calculate the absorption loss, and the f is the frequency of the acoustic wave. The *a*(*f*) can be expressed empirically, using Thorp’s formula as [12]:

(13) $$\{\begin{array}{c} 10\log a\left(f\right)=0.11\frac{f^{2}}{1+f^{2}}+44\frac{f^{2}}{4100+f^{2}}+0.000275f^{2}+0.003\;f\geq 0.4\\ 10\log a\left(f\right)=0.002+0.11\frac{f^{2}}{1+f^{2}}++0.11f^{2}\;f<0.4 \end{array}$$

where *a*(*f*) is in dB/km, and *f* is in kHz.

The transmission loss *TL* can be calculated as follows [11]:

(14) TL=SL+AL=d×10logr+r×10loga(f)

### 2.4. Multipath and Doppler Effect

The multipath effect is a phenomenon of a wave from the source node transmitted to the destination node via two or more paths. Moreover, under the right conditions, the two (or more) arriving signals may interfere at the destination node. In UWSNs, the multipath effect is more severe than that in WSNs. In the deep water environment, the medium is homogeneous and surface and bottom reflections may be neglected. But in the shallow underwater environment, the transmission distance is larger than the water depth; moreover, depending on the depth of the water, the factors (e.g., acoustic speed, temperature, salinity, turbidity) are different. Hence, the shallow water environment can be divided into many layers from surface to bottom. The acoustic wave can be transmitted and reflected in many layers, and then the multiple arrivals of the same signal may be received at the receiver, which causes a significant multipath effect.

In WSNs, movement of the sending node or receiving node contribute to the changes in the radio channel response. As a consequence, this will result in frequency shifting as well as additional frequency spreading, and this phenomenon is the Doppler effect. The magnitude of the Doppler effect is proportional to the ratio *a* = *v*/*c*, where v is the relative speed between sending node and receiving node and *c* is the speed of underwater acoustic wave. As discussed in Section 2.2.1, the speed of the acoustic wave is much slower as compared to the speed of the electromagnetic wave. Hence, the Doppler effect is more severe in UWSNs. This effect causes distortion in two ways: spreading the received signal bandwidth *B* to (1 + *α*)*B* which is referred to as the motion-induced Doppler spreading, and shifting the reception frequency *f* by an offset of *α* *f* which is referred to as Doppler shifting [13]. The Doppler effect and time synchronization can influence localization accuracy.

### 2.5. Transmission Error

As discussed above, the underwater acoustic channel is significantly affected by many factors such as water temperature, low speed of acoustic wave, ambient noise, transmission loss, multipath effect, and Doppler effect [2]. All these factors may cause delay variance and bit error, which result in high bit error rate and packet loss probability in UWSNs. Moreover, the underwater acoustic channel has the character of an open channel, which is shared by all nodes within the communication range. In this case, an attacker can passively intercept and analysis acoustic signals, and even worse actively disrupt network services such as localization, time synchronization and routing. Hence, it is a great challenge to design an effective secure protocol to protect UWSNs from eavesdropping and other malicious attacks.

### 2.6. Dynamic Network Topology

While terrestrial sensor nodes are densely deployed, underwater the deployment is deemed to be sparser due to the cost involved and to the challenges associated to deployment itself. The majority of underwater sensor nodes are mobile due to water flow. From empirical observations, underwater objects may move at the speed of 2–3 knots or 36 km/h in a typical underwater condition, which results in a highly dynamic network topology.

As discussed in Section 1, the cluster-based network architecture is widely used in UWSNs. Moreover, to expand the monitoring and communication region, AUVs are widely utilized in many applications. The AUVs may frequently join and exit the cluster or network, which will also result in a highly dynamic topology.

**Figure 2:**
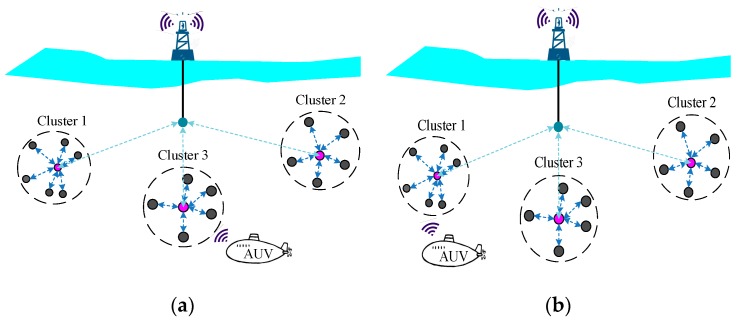
Cluster UWSNs with autonomous underwater vehicles (AUVs); (**a**) AUV joins Cluster3; (**b**) AUV joins Cluster1.

As shown in Figure 2a, to communicate with a surface statin or on-shore sink, the AUV joined Cluster 3 as a member node to transmit packets via Cluster 3. As shown in Figure 2b, due to the movement, the AUV was out of the communication range of Cluster 3, the AUV exited and then joined Cluster 1 as its member node. The movement of the AUV led to variation of the network topology.

As a consequence, these variations of the network topology mentioned above may change routing and influence the accuracy rate of data transmission which can affect the overall performance of the network. In particular, in some underwater applications with AUVs, due to the high mobility of AUVs, cooperation within the nodes and designing adaptive protocols can be a major challenge.

### 2.7. Insecure Working Environment

For some specific fields of application, for example, underwater security monitoring or target tracking, the working environment of UWSNs may be insecure. The underwater sensor nodes may be deployed to monitor hostile objects in high seas or hostile sea regions. These nodes could become highly vulnerable to threats and malicious attacks. These nodes may be physically destroyed and, even worse, the malicious attackers may compromise nodes to get the data and inject the compromised nodes into the network acting as legitimate node to cause continuous and more serious damage.

### 2.8. Physical Vulnerability

In general, UWSNs nodes are waterproof, compact and sophisticated in nature. As mentioned above, sensor nodes may be deployed at harsh and unattended sea regions. They could be physically damaged if struck, making them invalid, and are also vulnerable to marine organisms. It is impossible to guard each node from potential physical damage.

## 3. Threats and Challenges in Underwater Wireless Sensor Networks (UWSNs)

As discussed in Section 2, UWSNs and underwater acoustic channels suffer from many constraints that lead to potential safety hazards. As a consequence, UWSNs are vulnerable to various threats and malicious attacks. In this paper, these threats and attacks were discussed and analyzed in detail.

**Figure 3:**
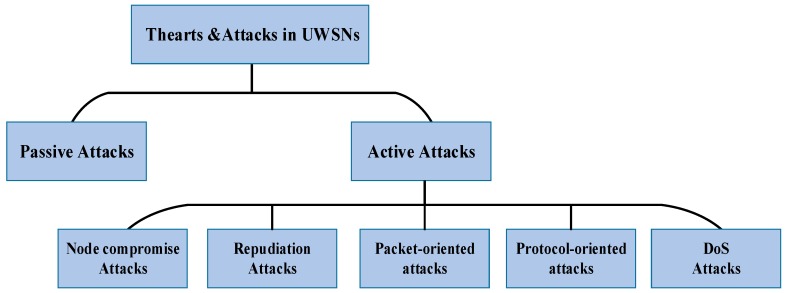
Threats and challenges in UWSNs.

According to actions taken by the malicious attacker, these attacks can be *passive* or *active*. As shown in Figure 3, these threats and attacks can be generally divided into two broad categories, *passive attacks* and *active attacks*.

### 3.1. Passive Attacks

Passive attacks refer to the attempts that are made by malicious nodes to perceive the nature of activities and to obtain data transmitted in the network without disrupting the operation. For example, eavesdropping, interfering, leakage of secret information, impersonation, message replay, and message distortion. As discussed in Section 2.2, the underwater acoustic channel is an open channel. Malicious attackers can easily capture packets transmitted in the channel by using a hydrophone or underwater microphone. Moreover, the attacker may predict the nature of communication by analyzing the packets traffic, observe the exchange of the packets, identify communicating hosts, and determine the location of nodes. Based on these actions, the malicious attacker can launch active attacks to cause more severe damage. Unfortunately, it is difficult to detect these passive attacks, since the network operation is not affected. To prevent this problem, the best solution is encryption mechanisms which make it hard for eavesdroppers to gain any information. Due to the large overhead and high energy consumption, the existing encryption mechanisms that used in WSNs and other wireless networks cannot be directly transplanted into UWSNs. The encryption mechanisms for UWSNs will be described in more detail in the subsequent sections.

### 3.2. Active Attacks

Active attacks refer to the attacks that attempt to alter, inject, delete or destroy the data transmitted in the network. Active attacks may intercept network data, and even worse, attempt to modify or drop packets to disrupt the communication and the operation of the network. Active attacks can be executed by internal or external malicious attackers. If the attacks are carried out by nodes that do not belong to the network, these kind of attacks are external attacks, which would be easier to detect and defend. Otherwise, if attacks come from an insider node, these kind of attacks are internal attacks, which can cause considerable damage to the network. It is unfeasible to detect a malicious node which is disguised as a normal node and then prevent it from disrupting the network. Even worse, internal attacks may be launched by compromised nodes which are actually legitimate nodes before being compromised. The compromised node has legitimate ID and other privacy data (e.g., secret key, encryption algorithm, trust value), which would act as a legitimate node and cause continuous attacks. From the analyses above, it is obvious that internal attacks are more difficult to detect and may cause more severe damages than external attacks. To prevent this problem, the feasible solution is using security mechanisms such as encryption, authentication and trust management. These security mechanisms suitable for UWSNs will be described in more detail in Section 4.

According to the intention of attacks, active attacks can be classified in the following categories: node compromise attacks, repudiation attacks, packet-oriented attacks, protocol-oriented attacks, and denial of service (Dos) attacks [14].

- *Node compromise attacks*: a malicious attacker can tamper with underwater sensor nodes physically. As discussed in Section 2, in some specific fields of applications, underwater sensor nodes may be deployed in unattended and even worse hostile sea regions. Moreover, the network may consist of tens or hundreds of nodes deployed in large scales, which means that it is unable to ensure the safety of all nodes. An attacker can locate the nodes by monitoring the intensity of the acoustic signal and capture them. Worse, if there are no hardware hack-proof technique or other security mechanism, the attacker may easily crack and compromise them to read privacy data (e.g., secret key, encryption algorithm, trust value) and modify these data from internal memory. Moreover, the compromised node may be injected into the network as a legitimate node to monitor or cause continuous attacks. Therefore, in the vast majority of cases, the node compromise attacks are combined with other active attacks to cause more severe damage to disturb communication and cooperation between sensor nodes. To prevent network from these attacks, hardware hack-proof techniques, re-configuring, and trust management mechanisms should be designed and used for UWSNs.
- *Repudiation attacks*: in repudiation attacks, malicious nodes deny having any involvement in particular action or communication with other nodes. This refers to the denial by a node involved in a communication of having participated in all or part of the communication, regardless whether that communication is malicious or not.
- *Packet-oriented attacks*: in packet-oriented attacks, the malicious adversary lunches attacks that aiming at disrupt packet transmission or destroy the data of the packet. There are some common active attacks including: interception attack, modification attack, and injection attack. An interception attack is to capture packets from an acoustic channel by intercepting. Moreover, the attacker can read and modify the content of the intercepted packets which is called a modification attack. An injection attack is to inject useless or harmful packets into the network to consume nodes’ energy or disrupt the network.
- *Protocol-oriented attacks*: the malicious adversary launches attacks that aiming at disrupt the function of some specific protocols. The main categories of this kind of attacks are: routing protocol attacks and media access control (MAC) protocol attacks. Routing protocol attacks can cause packets unable to be transferred to the destination node, and even disrupt the operation of the network. These types of attacks are mounted on the routing protocols, such as routing table overflow, routing table poisoning, packet replication, and rushing attacks. Through these malicious behaviors, attackers can attract packets and analyze or even drop packets at its will. MAC protocol attacks aimed at disrupting the mechanism that control nodes access to channel. Malicious attackers can continuously occupy the channel to deprive legitimate nodes’ chances of sending packets. Moreover, for the request to send/clear to send (RTS/CTS) handshake MAC protocol, continuously sending RTS packets would consume nodes’ battery which is a cheap and easy way to lunch attacks. To defend against these attacks, the feasible solution is using encryption, authentication and trust management mechanisms suitable for UWSNs.
- *DoS attacks*: DoS attacks attempt to make resources and services unavailable to the legitimate nodes. To achieve this goal, the attacker tried to prevent legitimate nodes to access services offered by the network. DoS attacks can be passive or active, and can be carried out in many different ways. Combined with other passive or active attacks, it is more difficult to detect and defend against these attacks.

Among these active attacks discussed above, DoS attacks are more destructive, complicated, and hard to detect [15]. To guard UWSNs from DoS attacks, the approaches and features should be comprehended.

### 3.3. DoS Attacks

DoS attacks can be launched in different ways that do not exist in other wired or wireless networks and which can be launched at any layer of the protocol stack [16,17]. Even if UWSNs are well protected by encryption technology, they can still be threatened by DoS attacks, which can disrupt communication and cooperation between nodes and decrease availability of the whole network. Moreover, Dos attacks are low cost, deadly, and even worse, hard to detect and defend. Malicious attackers can cause severe damage at very low cost, impersonate as a legitimate node to deceive neighbor nodes, or impose particularly high power cost tasks to legitimate nodes to shorten the nodes lifetime. According to the UWSNs network model, DoS attacks and the corresponding defense technologies are discussed as below.

#### 3.3.1. Physical Layer

The physical layer of UWSNs is in charge of the acoustic communication between nodes in the network. As discussed in Section 3.1 Passive Attacks, the malicious attacker can eavesdrop transmissions, and then predict the nature of communication by analyzing the packets traffic, observe the exchange of the packets, identify communicating hosts, and determine the location of nodes. With this information, the attacker can perform several DoS attacks in the physical layer, including *eavesdropping attacks* and *jamming attacks*.

##### Eavesdropping Attack

An eavesdropping attack is a kind of passive DoS attack that listens for acoustic transmissions between nodes and captures packets transmitted in the channel. If the packets are not encrypted, the attackers could easily read the data and privacy information. As a consequence, it is difficult to detect if eavesdropping is actually occurring since the attacker does not disrupt the normal communication.

In [18], the authors presented an analytical model which investigates the probability of eavesdropping attacks with consideration of underwater acoustic channel conditions, including signal attenuation and ambient noise. The relationship between the eavesdropping success condition and the underwater acoustic signal channel is established. The authors derive the eavesdropping probability from consideration of both isotropic eavesdropper and array eavesdropper, respectively. Moreover, the probability heavily depends on the acoustic signal frequency, spreading factor, wind speed and node density.

##### Jamming Attack

**Figure 4:**
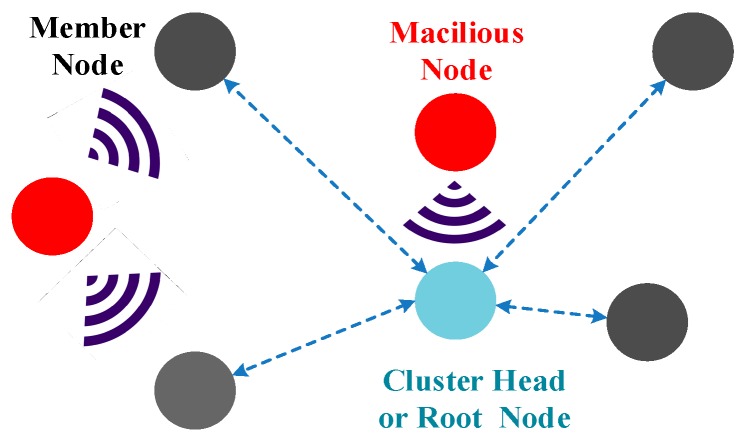
Jamming attack.

As shown in Figure 4, in a jamming attack, one or several malicious nodes constantly emit useless or undesired signals, and thus normal communication with other legitimate nodes in the affected region will be interfered with. Worse, the malicious nodes can paralyze the entire network by jamming only a few special nodes such as cluster-head node, root node or base station. These special nodes are responsible for aggregating data, transmitting packets, and managing the network. For example, as shown in Figure 1, the underwater sink node aggregates data from sensor nodes and then sends this data to the on shore base station or surface sink. Jamming attackers can continuously send packets or communication requests to the underwater sink node. In this manner, the underwater sink node is not able to respond to requests from legitimate nodes or receive packets. Moreover, the attacker may simply keep requesting an opportunity to send data to realize the goal. A jamming attack can effectively disrupt localization protocols in an underwater environment.

In [19], the author defined two types of attackers with four different attack methods and introduced the effects of jamming attacks on underwater acoustic networks (UANs). These attacks are evaluated on three commonly used underwater acoustic modems in a real-world experimental testbed in Mansfield Hollow Lake, Mansfield, Connecticut and in a lab testbed. But no feasible solution to defend jamming attack is given in this article.

To defend against jamming attack, the majority of anti-jamming mechanisms used in WSNs cannot be directly used in UWSNs. Spread-spectrum techniques are widely used in underwater acoustic communication. Among these techniques, the frequency hopping spread spectrum (FHSS) and direct sequence spread spectrum (DSSS) are drawing interest for their good presentation over noise and multipath interference [20]. To an extent, FHSS and DSSS techniques are resistant to interference from jammers. Even if a FHSS technique is used, the jamming attacker can jam a wide band of the spectrum. Worse, the DSSS technique is also vulnerable to a high-power jamming signal. Another way to defend against a jamming attack is that the nodes switch to sleep mode and periodically wake up to check the attack is over. Unfortunately, for some kind of UWSNs, these spread-spectrum techniques are not suitable. In [21], a multi-armed bandit–based acoustic channel access algorithm is proposed to achieve the jamming-resilient cognitive acoustic communication. In [22], the author resorted to jamming-resilient techniques such as multipath transmissions. The results showed that the inherent redundancy of multi-path routing offers an effective shield against excessive packet losses in the presence of strong jamming. In [23], a detection scheme based on a partial-packet for reactive jamming is proposed. The work estimated the probability of high deviation in received signal strength (RSS) using a weak estimation learning scheme. The performance was evaluated through simulation, which showed that the proposed scheme is capable of accurately detecting reactive jamming in UWSNs. In [24], the authors studied the characteristics of jamming in UWSN, and proposed an underwater jamming detection protocol (UWJDP) to detect and mitigate jamming in underwater environments. The results showed that if the packet delivery ratio is less than or equal to 0.8, the mechanism will reach the maximum probability of detecting jamming.

#### 3.3.2. Datalink Layer

Due to the limited bandwidth of the underwater acoustic channel, MAC protocols are utilized to enable multiple devices to share the acoustic channel in an efficient and fair way. But unfortunately, there are several kinds of DoS attacks that can be launched at this layer to consume the battery of legitimate nodes or disturb normal MAC operation, including the *Jamming Attack*, *Collision Attack*, *Exhaustion Attack*, *Denial-of-sleep Attack*, and *Unfairness*.

##### Jamming Attack

A datalink layer jamming attack is similar to that in the physical layer, but in a smarter and more efficient way. The malicious attacker can achieve the goal by just repeatedly sending request to send (RTS) packets. The legitimate nodes are deprived of the chance of accessing the channel. As a consequence, this disrupts the cooperation between nodes and communication of the network. In contention-based MAC protocols, the malicious attacker can give itself the highest priority and occupy the channel all the time. Therefore, scheduled MAC protocols are capable of defending against the attack. Anti-replay protection and link-layer authentication can mitigate these attacks. Unfortunately, receiving large quantities of RTS packets still consume a node’s energy and occupy channels.

##### Collision Attack

For the RTS/CTS handshake mechanism, in which any node detected either an RTS or a CTS packet from the channel, it should not transmit packets during the time period. However, a malicious node may violate this mechanism, and transmits packets even after detecting a CTS packet destined for a legitimate node, which will cause a collision at the receiver. It is obvious that a change will occur in the collisional data, and the packet will become invalid. Colluding in a collision attack interrupts packets during communication, and a mitigating colluding collision technique can defend against a colluding collision attack [25]. In a manner, an error correcting code is a feasible technique to avoid collision.

##### Exhaustion Attack

An exhaustion attack refers to keeping the channel busy and exhausting the node’s battery by introducing a malicious node in the network. It can be launched by the intruder, or by a compromised node on which the internal program code was modified by an intruder. Another form of exhaustion attack is that the intruder node sends numerous join-requests or RTS/CTS packets to force the target node to send or receive. The feasible solution is rate limitation on each node in the network. A fuzzy logic based solution against distributed node exhaustion attack is proposed in [26].

##### Denial-of-Sleep Attack

A denial-of-sleep attack is to prevent the node from going into sleep mode [27]. An attacker might choose to execute a denial-of-sleep attack over a simple jamming-based DoS attack. To permanently disable the network, it may take months to exhaust the targeted node’s batteries. A smarter denial-of-sleep attack that keeps the sensor nodes on working mode would drain the batteries in a very short period (maybe only a few weeks or days). Worse, some denial-of-sleep attackers are not required to constantly send signals, making it more difficult to locate the attacker via its emitted transmissions.

##### Unfairness

This is a weak form of DoS attack which is performed by attacker to degrade the network performance instead of completely preventing legitimate nodes from accessing the channel. A small frames technique can be used to reduce the amount of time. This technique lessens the effect at the expense of efficiency. Moreover, it is susceptible to further unfairness. For example, an attacker may retransmit at a higher speed instead of randomly delaying.

The majority of datalink layer DoS attacks discussed above can be prevented by using error detection code, rate limitation, and by dividing the packets into small frames.

#### 3.3.3. Network Layer

Network layer is responsible for routing packets from the source node to the destination node. Owing to the particularities and constraints discussed in Section 2, the network layer is vulnerable to many threats and attacks which aim to disturb the routing of the network, including the *Replay Attack*, *Selective Forwarding Attack*, *Neglect and Greed*, *Misdirection Attack*, *Black hole/Gray hole* *Attack*, *Sinkhole Attack*, *Wormhole Attack*, *Sybil Attack*, *Hello Flooding Attack*, and *Homing* *Attack.*

##### Replay Attack

In a replay attack, the attacker (A) intercepts a message sent by sender (S) to receiver (R), after a short delay, then resends it to R. Even though the packet is received, but the arrival time at R is changed due to the delay by A. As a consequence, the fake propagation time and signal strength result in R receiving imprecise locations and distance between them, which is usually estimated according to signal arrival time or difference in signal strength. Authentication and anti-replay techniques can protect against a replay attack.

In [28], an underwater media access control protocol based on the RTS/CTS mechanism with a cipher block chaining-message authentication code is proposed to provide data confidentiality, authenticity, and replay attack protection. In [29], a secure MAC protocol for cluster-based UWSNs is proposed to ensure the security of data transmission. A random nonce is used to prevent a replay attack, which is updated each time after the message was transmitted. Therefore, the attacker cannot pass the authentication even if the attacker had made a copy of the previous message.

##### Selective Forwarding Attack

In this attack, malicious nodes behave like legitimate nodes, but selectively drop packets, and refuse to forward the received packets. However, there is a risk that the neighbor node may find other paths to route the packet to the destination node. Hence, in order to avoid being detected it selectively forwards certain packets and drops certain packets. The attacker interested in suppressing and modifying a packet originating from few selected nodes can reliably forward the remaining and limit suspicion of its wrongdoing [30].

To detect a selective forwarding attack, pre-defined watchdog mechanism can be used for WSN, which have pre-defined rules for raising intrusion alerts. In this mechanism, attacks can be detected by allowing nodes to listen to the next hop nodes in a broadcasting transmission [31]. Unfortunately, the pre-defined watchdog mechanism is unsuitable for UWSN. This mechanism will fail in the case of a collision due to uncertainty ambiguity in the receiver, inadequate transmission power, fake misbehavior, and partial packet dropping. In [32], evidential evaluation is used to identify the selective forwarding attacks, which utilize the Dempster–Shafer theory of combined multiple evidences. Hop-by-hop cooperative detection scheme was proposed in [33] to detect and mitigate the selective forwarding misbehaviors for WSN. The trust management and reputation mechanisms based on behavior evaluation can be used to detect these attackers and then isolate them from the network [34,35].

##### Neglect and Greed

This attack is a special case of selective forwarding attack, as the malicious attacker may randomly drop the received packets but still acknowledge the source node (Neglect attack), or give excessive priority to its own packets (Greed attack). The protocols which are based on dynamic source routing are the most vulnerable to this attack [36].

The feasible solution to this kind of attack is to declare alternative routing paths, and using redundant messages is another feasible solution. But in exchange, more energy will be consumed, which is the most severe resource constraints for UWSNs.

##### Misdirection Attack

**Figure 5:**
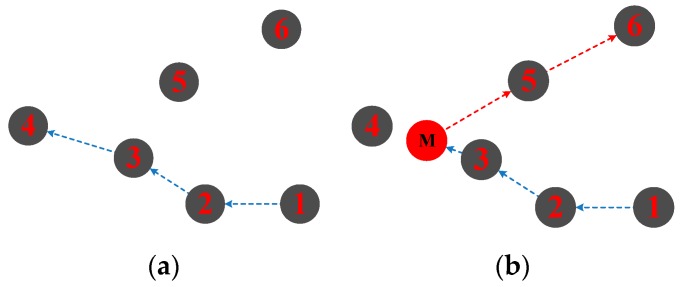
Misdirection attack; (**a**) normal route; (**b**) misdirection route.

In a misdirection attack, the malicious attacker forwards packets to incorrect paths by modifying routes or misdirecting packets to a malicious node. As illustrated in Figure 5a, the normal route should be from Node1 to Node4, but the malicious attacker (M) modified the route to Node6.

This attack can be protected by amending the route path which consists of source-routes in each packet. Authorization, egress filtering, trust-aware routing, and monitoring of routes are feasible techniques for defending against a misdirection attack. In [37], a defense mechanism is proposed for the detection and isolation of the misdirection attack in WSNs. The proposed technique is based on node localization, in which delay per hop is counted. The node that is increasing delay maximum times would be detected as a malicious node. Unfortunately, it is hard to get nodes’ precise positions and the distances between nodes in the underwater environment, which means that the mechanism is not suitable for UWSNs.

##### Black Hole/Gray Hole Attack

In this attack, an attacker acts as a black hole by broadcasting forged routing information with the lowest cost or shortest path toward destination. The victim nodes would select this path as the optimum route which actually goes through the attacker. As a consequence, the attacker can attract and pull in all the traffic of the network. Moreover, the attacker may analyze, modify, or even drop packets at will. If the attacker drops all data packets, this attack is known as a black hole attack. If it selectively drops some critical packets, it is known as a gray hole attack.

**Figure 6:**
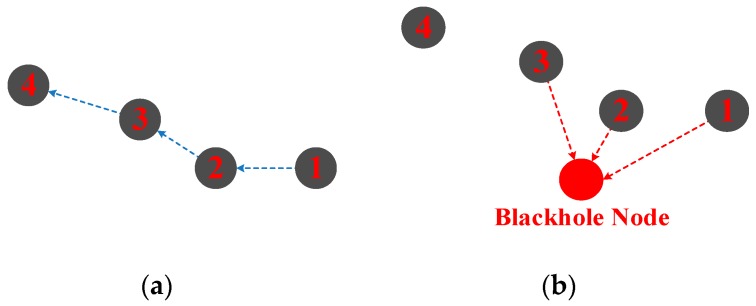
Black hole attack; (**a**) normal route; (**b**) black hole route.

As illustrated in Figure 6a, based on the location information, the packets from Node1 will be relayed by Node2 and Node3 to Node4. A black hole node will broadcast its false location to disguise itself as the node nearest to the sink node. As illustrated in Figure 6b, nodes in the network mistake the black hole node as the best choice to relay packets to the sink node. Hence, all the packets would be captured by the black hole node.

This type of attack is harmful for sensor nodes that are deployed considerably far from the sink node. In a smarter way, the attacker may drop critical packets during a certain time period or with a certain percentage, making it more difficult to be detected. Some existing work has addressed this attack and feasible countermeasures have been proposed. In [38], an efficient and trust-based distributed intrusion detection system is proposed to defend against single and cooperative gray hole and black hole attacks. A secure routing protocol based on elliptic curve cryptography (ECC) is proposed in [39] to detect and defend false reports and gray hole attacks. A trust-based clustering protocol is proposed to detect the gray hole and prevent compromised nodes from becoming cluster head [40].

##### Sinkhole Attack

**Figure 7:**
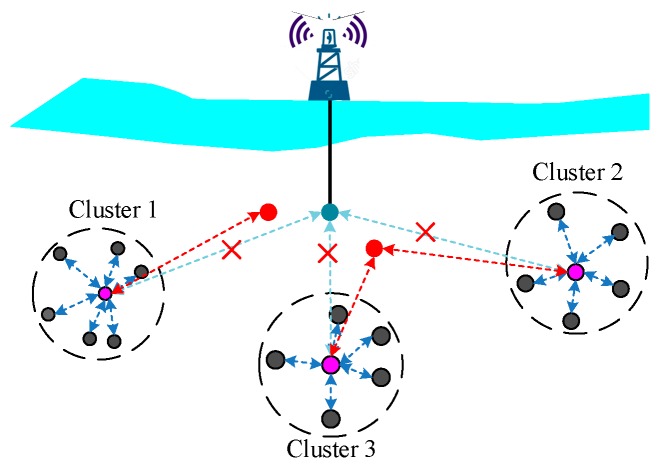
Sinkhole attack.

The sinkhole attack is a particular black hole attack that prevents the legitimate sink node from obtaining data transmitted by underwater sensor nodes, and it will cause serious threats to higher-layer protocols and applications. To gain the goal, the attacker manages to attract almost all traffic that is destined to the sink node, which appears more attractive to neighboring nodes by disguising them as the shortest hop count path. Then, the attacker may drop the packets or get privacy data from them. As illustrated in Figure 7, the ideal location for such an attacker is near the legitimate sink node.

AODV (Ad hoc On-demand Distance Vector Routing)-based secure routing algorithm with mobile agent is proposed to detect malicious node in network [41]. In [42], an intrusion detection system (IDS) against sinkhole attacks is proposed, in which the network area is divided into a flat grid of cells. The signature-based technique was used to detect and remove fake sink nodes. The proposed IDS considered two types of sink mobility: periodic and random. Geographic routing [43,44] and authentication are feasible countermeasures against this attack. Unfortunately, geographic routing protocols are also a challenging research topic in UWSN.

##### Wormhole Attack

**Figure 8:**
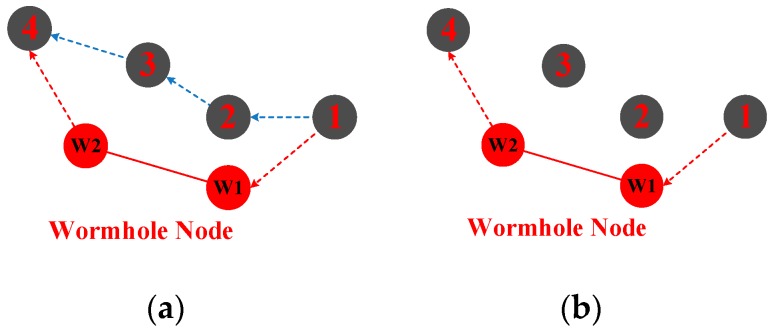
Wormhole attack; (**a**) normal route; (**b**) wormhole route.

In a wormhole attack, two distant malicious nodes are commonly involved, which constitute a wormhole attack tunnel. As illustrated in Figure 8a, A malicious attacker (labeled as W1) captures packets from source node N1 and uses a faster tunnel to send the packets to another attacker node (labeled as W2) which delivers the packets to the destination node N4.

**Figure 9:**
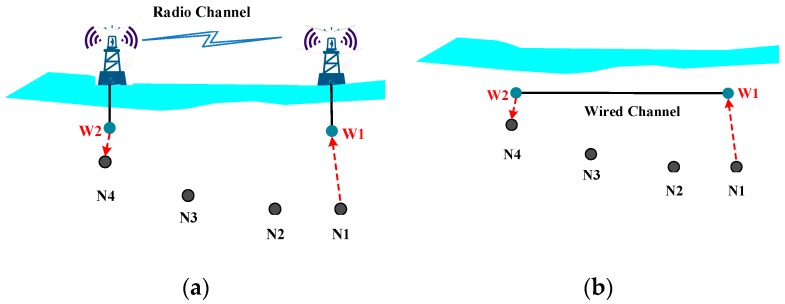
Two methods of wormhole attack; (**a**) using radio channel; (**b**) using wired channel.

As shown in Figure 9, the wormhole tunnel can be a RF link (above the water surface) or wired link, which is much faster than underwater acoustic channel [45]. This attack can prevent the source node from discovering and selecting other legitimate routes and thus disrupts network functionality. Similar to the black hole attack, a wormhole attack can attract packets forwarding and severely disturb normal routing. Wormhole attack detection and countermeasures have attracted many research studies. In [46], a wormhole attack was simulated in NS 2. The work selected the existing wired object link in the NS 2 wired simulation package to simulate the “tunnel” by copying the local packets and directly sending them to the wormhole object installed in a remote node. A secure localization algorithm for UWSNs is brought out in [47], which is based on the constraints of propagation distance and reputation values. The anchor nodes evaluated the reputation of paths to other anchor nodes and broadcast these reputation values to the network, and unknown nodes select credible anchor nodes with a high reputation. In [48], a two-tier localization scheme is proposed to identify short-range wormholes instantly, and long-haul wormholes within a limited latency. A distributed approach to detect and mitigate wormhole attacks is identified in [49], and an analytical model is provided to capture the interactions between various contributing parameters. In [50], a distributed visualization of wormhole is proposed to detect wormhole attacks. However, it is not feasible for large-scale or high-density UWSNs, since the reconstruction of the network would cause large energy consumption.

##### Sybil Attack

In the Sybil attack, an attacker may forge multiple identities and pretend to be in multiple places at the same time. The basic goal of these false identities is to deny the information passing procedure. These multiple identities can be occupied by fabricating faults or stealing the identities of legitimate nodes. Hence, the Sybil attack can also cause severe damage to distance-based or location-based routing protocols. Moreover, the attacker can behave as a base station or receiver, which sends acknowledgment packets to sensor nodes to avoid retransmission.

Authentication and position verification are feasible countermeasures, but position verification is difficult owing to mobility. The author of [51] presented a robust and lightweight scheme to detect a Sybil attack, which is based on received signal strength indicator (RSSI) readings of messages. Another countermeasure is the random key pre-distribution [52], which relies on cryptographic principles and is easy to analyze. In [53], a Sybil attack detection scheme based on state information of the nodes is proposed, which assume the availability of beacon nodes. But, this scheme assumes that each node is stationary and has the same transmission range over bidirectional links, and depends on the density of legitimate neighboring nodes.

##### Hello Flooding Attack

In some routing protocols of UWSNs, nodes broadcast hello messages to inform the presence to one-hop neighbors. An adversary may launch attacks by recording the hello packets and sending them from a super node with higher transmit power and larger communication range. These replayed hello packets can reach nodes which are out of communication range of the source node. Any node that uses the source node as the next hop in a route but that is out of that node’s communication range will be unable to forward packets.

Pairwise authentication and geographic routing protocols are feasible countermeasures for this attack. In pairwise authentication schemes, nodes can verify bidirectional links before constructing routes. Geographic routing protocols [54] that let nodes discount hello messages from nodes without communication range can also prevent UWSN from this attack. Geographic protocols require each node to know its location and be able to communicate that location to other nodes. However, precise positioning is also a challenging research topic in UWSNs.

##### Homing Attack

In a homing attack, malicious attacker may analyze the traffic to identify and target nodes that have special responsibilities, such as cluster heads or sinkhole node. Moreover, the attacker may launch other DoS attacks to jam or destroy these special nodes.

Header encryption is a common countermeasure, but it does not completely prevent analyzing traffic. It might be enough to identify the location of these special nodes by simply analyzing the volume of traffic in various portions of the network. In [55], an anti-traffic analysis strategy is proposed to help disguise the location of the base station from eavesdroppers by using “dummy packets”. Unfortunately, these dummy packets significantly consume nodes’ energy, especially for UWSNs. Hence, it can be used only when it is of utmost importance to prevent traffic analyzing.

#### 3.3.4. Transport Layer

The transport layer of UWSNs is responsible for the reliable transport of packets. Typical DoS attacks at this layer include the *desynchronization attack* and *synchronization flooding attack*.

##### Desynchronization Attack

In a desynchronization attack, a malicious attacker interrupts active connections between nodes by transmitting forged packets with bogus sequence numbers or control flags that desynchronize endpoints. For UWSNs, synchronization is very important and difficult, moreover, the global positioning system (GPS) is not suitable [2]. Hence, GPS-free synchronization mechanisms are used. A malicious attacker may forge messages carrying wrong sequence numbers to disturb synchronization between nodes, which can affect the accuracy of the synchronized clocks and the efficiency of scheduled operations. Worse, all GPS-free time synchronization schemes are vulnerable to wormhole attacks. If the attacker combined a desynchronization attack with a wormhole attack, it could severely disrupt communication and cooperation of the whole network. For example, wormhole, Sybil, or replay attacks may cause fake measurement results on ranges or round-trip time (RTT) between legitimate nodes, which are important parameters for time reference alignment. Header or full packet authentication can defeat such an attack.

##### Synchronization Flooding Attack

When a protocol is required to maintain a state it becomes vulnerable to memory exhaustion through flooding. An attacker may repeatedly make new connection requests until the resources required by each connection are exhausted or reach a maximum limit. A very common form of DoS attack involves sending a large number of common packets, e.g., transmission control protocol (TCP), internet control message protocol (ICMP), and user datagram protocol (UDP), which aimed at a single destination. The huge traffic deluge caused by these packets leads the network to no longer be able to distinguish between legitimate and malicious traffic.

Using connectionless transport protocols is a feasible countermeasure, but at the expense of lack of necessary transport-layer functionality to applications. The primary defense against this attack is synchronize sequence numbers (SYN) cookies [56], which encode information from the client’s TCP SYN message and return it to the client to avoid maintaining state at the server. Unfortunately, this technique calls for high computational capacity and causes extra messages overhead, which makes it undesirable for UWSNs.

## 4. Security Issues of UWSNs

### 4.1. Security Requirements

As discussed in Section 3, UWSNs are vulnerable to various attacks. Hence, UWSNs should meet some security requirements in actual applications. As a branch of WSNs, the security requirements of UWSNs are similar to terrestrial WSNs [7]. But due to the particularities and constraints of UWSNs discussed in Section 2, there are also some special security requirements.

(1) Confidentiality

This concerns preventing unauthorized nodes from understanding the contents of the sensitive data (e.g., security credentials and secret keys). Confidentiality is not restricted only to the survivability of a user’s information (e.g., strategic or tactical military information), but also to the survivability of the MAC, routing information, etc. These sensitive data should be prevented from reading or tampering by a malicious attacker. Confidentiality can be achieved by applying a low power-efficient encryption technique which is suitable for UWSNs. The cipher text stealing technique [57] is a lightweight typical encryption technique used in UWSNs.

(2) Authentication

As discussed above, the acoustic channel is open and, moreover, the malicious attacker can easily capture and modify packets if there is no encryption technique. Hence, receiving node needs to identify the source of the data in order to filter malicious attacks. Only authorized nodes have the right to access and share channel, services, applications and data on that network. Intrusion detection and a trust management mechanism can be utilized to identify abnormal activities to remove malicious nodes from the network. These mechanisms ensure that only the authorized nodes have permission to perform in the network.

(3) Integrity

Data integrity is to ensure that the received data is not modified, removed, or corrupted in transition by unauthorized nodes either by radio failure or malicious attack. This is most essential in circumstances such as military operations and equipment controls where such changes could cause serious damage. The message authentication code [58] for data authentication has been widely applied in WSNs and UWSNs, which has good scalability, low latency, reliability, adaptability and ease of implementation.

(4) Freshness

Freshness is to ensure that the received data is fresh and it is not the retransmission of legacy data. Routing updates should be delivered in real time. The delay of the update messages might reflect the wrong state of the network and lead to a large loss in information.

(5) Availability

Availability is to ensure that the network must be robust enough. Even if some nodes fail or the system is attacked, it will still be able to provide services. Proper redundancy tactics and self-adaptive tactics can supply availability for UWSNs.

(6) Isolation

Isolation is to ensure that nodes should be able to identify abnormal activities and isolate malicious nodes. Moreover, MAC protocols and routing protocols should be immune to malicious attacks. Proper trust management and lightweight cryptography algorithms can be used to isolate malicious nodes.

(7) Self-stabilization

Self-stabilization is to ensure that nodes should be able to recover from attacks independently in real time without intervention. If a node is self-stabilizing to malicious attacks, it can automatically recover its normal state, even if the attacker remained in the network.

(8) Survivability

This is the capability of the system to fulfill its mission in a timely manner, in the presence of accident, failure, intrusion or malicious attacks. It is to ensures that the network can restore and maintain essential services during and after malicious attacks, even if part of the network had been destroyed.

### 4.2. Security Mechanisms

To achieve the objectives of the security requirements mentioned above, a set of security mechanisms and technologies should be utilized to enforce these security requirements and prevent UWSNs from attacks.

**Figure 10:**
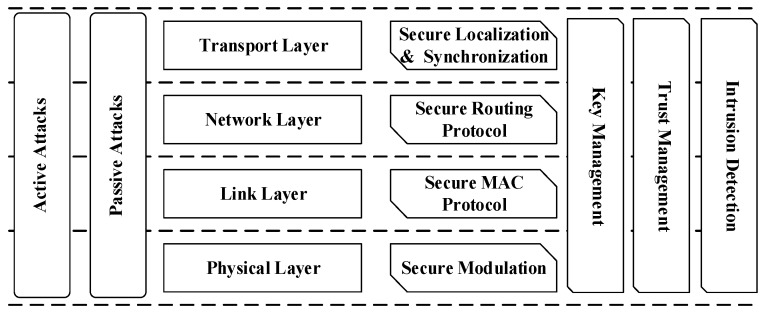
Security architecture of UWSNs.

To provide a comprehensive solution, according to the open system interconnection (OSI) network, the security issues of UWSNs are logically divided into separate secure components. As shown in Figure 10, the security architecture of UWSNs can be divided into four layers: physical layer, link layer, transport network layer, and application layer.

The security issues that are applied in UWSNs include: key management, intrusion detection, trust management, secure localization, secure synchronization, secure MAC, and secure routing.

#### 4.2.1. Key Management

Cryptographic and key management play very important roles in ensuring confidentiality, authentication, integrity, and non-repudiation. Cryptography enables sensitive information to be stored or delivered in unsecure networks such as the underwater acoustic channel so that it cannot be read or modified by unauthorized users. Due to the particularities and constraints of UWSNs discussed in Section 2, the encryption and key management mechanisms for WSNs are unsuitable for UWSNs. In these traditional public key cryptography (PKC) schemes, a public key infrastructure (PKI)-based method needs the certificate authority (CA) to manage the certificate and key distribution, which result in high communication and computation cost. It is not affordable for resource-constrained UWSNs [59,60]. In identity-based encryption (IBE) schemes, there is no need for PKI, and the public keys are computed according to the node ID. Hence, an IBE scheme avoids the resource cost for storing and granting certificate, which makes it a feasible scheme for public key encryption in WSN. However, distribution of a private key by PKG will cause high communication costs, which is a heavy burden for UWSNs.

Related research has been presented for UWSNs in recent years [61,62,63,64,65,66,67,68]. Unfortunately, the existing cryptography and key management mechanisms are suffering from some problems, including cipher text expansion and computational complexity.

Message padding and codes increase the length of messages after applying cryptography and cause more energy consumption on transmission and computation. For instance, with the standardized AES encryption, the block size is 128 bits, and the message expansion due to padding is around 18% for a typical UWSN message of 720 bits [69].

A digital signature is usually used for message authentication. A digest is appended to an authenticated message that will cause expansion and communication overhead. For example, the size of a digest produced by SHA-256 is 256 bits, which can cause an overhead up to around 35% of an average UWAN message [70].

#### 4.2.2. Intrusion Detection

Intrusion detection mechanisms are to detect, identify and isolate malicious attackers from the network, including internal or external intruders. However, intrusion detection mechanisms usually work after the malicious attacks take effect and have been discovered. It is difficult to detect malicious intruders the first time that attacks occur. Hence real-time detection mechanisms need to be researched and improved. Alternatively, intrusion tolerance mechanisms can be used to protect networks while allowing the existence of malicious intruders, which is considered to be an efficient security mechanism. Moreover, algorithms, technologies and IDS have been proposed to further improve UWSNs security.

*Standalone IDS*: there is no data exchanged between nodes; each node runs IDS and detects attacks independently.

*Distributed and Cooperative IDS*: every node participates in intrusion detection by having local and global detection decision-making.

*Hierarchical IDS*: this is suitable for multilayered UWSNs. Cluster head nodes in clusters perform the task of IDS and act as checkpoints such as routers in wired networks.

Among these types of IDS, the hierarchical IDS is suitable for UWSNs which are based on a cluster structure.

#### 4.2.3. Trust Management

As an important complement to security defense, a cryptography-based trust management mechanism has significant advantages in intrusion detection. Due to the particularities and constraints of UWSNs discussed in Section 2, the research on trust management mechanisms in UWSNs faces challenges [71].

The existing trust management mechanisms can be classified into three categories: centralized scheme, distributed scheme, and hierarchical scheme.

*Centralized Scheme*: in a centralized scheme, a root node or a base station supply trust management for each node in the network. The centralized schemes are inappropriate for UWSNs, because the energy consumption of trust values exchanging between sensor nodes and the base station is an expensive burden.

*Distributed Scheme*: in a distributed scheme, each node needs to compute and maintain the trust values of entire network. But it is impossible for UWSNs, as discussed in Section 2, as underwater sensor nodes are extremely limited in hardware resources. Hence, the distributed schemes are also inappropriate for UWSNs.

*Hierarchical Schemes*: this kind of trust management scheme is used by the PKI. There is a root CA which is directly trusted. The CA may certify certificates themselves, or may certify certificates that certify other certificates (trusted introducer) down some chain. The hierarchical scheme is represented as a tree structure. The leaf certificate’s validity is verified by tracing backwards from its issuer to other issuers, until a directly trusted root (CA) certificate is found.

As discussed above, it is obvious that neither pure centralized nor pure distributed schemes are suitable for UWSNs. In hierarchical schemes, the computation and transmission of trust value is implemented in a hierarchical way. The trust values are passed and merged from a lower layer to an upper layer. Therefore, the hierarchical schemes are more appropriate for cluster-based topology which was widely used in UWSNs. In order to broadcast control information and retrieve the readings from underwater sensor nodes, the sink node must be able to authenticate itself. Moreover, in most applications the sink node acts as an interface between the UWSN and the land network.

#### 4.2.4. Localization Security

Location estimation is a vital component in the source detection and tracking applications. The underwater sensor nodes get the location information and speed of mobile nodes during the localization phase. The location and motion information would be used in the routing protocol to select the best relay node to forward data. Without the location information, the sink node cannot identify where the received data comes from.

Due to the characteristics of the underwater channel (long propagation delays, Doppler effect, limited bandwidth, node mobility and multipath), localization protocols proposed for WSNs cannot work in underwater applications. With regard to the mechanisms used for location estimation, underwater localization algorithms can be classified into three categories: range-based schemes, range-free schemes, and hierarchical schemes [72]. In the range-based location algorithms, distance or angle estimates with neighbors will be used for calculating node locations. In the range-free location algorithms, due to the energy consumption and hardware limitation, the neighbor distances or angle information are assumed to be unavailable for positioning. The hierarchical schemes consist of four types of nodes, which are surface buoys, detachable elevator transceivers (DETs), anchor nodes and ordinary nodes. A surface buoy is assumed to be equipped with GPS on the water surface. A DET is attached to a surface buoy and can rise and down to broadcast its position. The anchor nodes can compute their positions based on the position information from the DETs and the measurement of distance to the DETs.

Some localization-specific attacks e.g., Sybil attack, black hole attack and wormhole attack can cause great damage by utilizing or modifying the localization information. Most of the existing localization protocols do not take security issues into account when being designed. The secure localization scheme should be able to determine the location of sensors even in the presence of Sybil and wormhole attacks, and the scheme should be able to node mobility in UWSNs. To defend against injecting false localization information in UWSNs, effective and efficient cryptographic algorithms need to be developed [73,74,75,76].

#### 4.2.5. Synchronization Security

As discussed in Section 4, synchronization is essential in many underwater applications and scheduling MAC protocols. As discussed in Section 2, due to the characteristics of UWSNs, synchronization security protocols proposed for WSNs are unsuitable for UWSNs. Moreover, it is especially difficult to achieve precise time synchronization in underwater environments.

Although it is critical among the UWSN issues, none of existing time synchronization schemes [77,78,79] take security in consideration. Proper cryptographic techniques can be used to defend time synchronization attacks (e.g., masquerade, replay and manipulation attacks). However, the countermeasures against delay attacks for WSNs [80,81,82] are not applicable to UWSNs.

#### 4.2.6. Routing Security

Routing security consists of basic transports and connectivity security mechanisms which are applied to routing protocols as well as the individual nodes. Moreover, nodes must exchange information with their neighbors to construct the network topology in order to apply one of the routing protocols (proactive, reactive and hybrid).

Routing security involves two aspects: secure routing and secure data forwarding. In secure routing, nodes are required to cooperate in order to share correct routing information, thus keeping the network connected efficiently, whereas in secure data forwarding, data packets must be protected from tampering, dropping, and altering by any unauthorized party. In recent years, many research papers have been presented to supply routing security for UWSNs [83,84,85,86,87,88].

### 4.3. Security Systems

With the development of technology, security issues have been taken more seriously. Much research on specific security issues has been undertaken, and moreover, relatively mature security systems for WSNs have been proposed. To the best of our knowledge, security protocols for sensor networks (SPINS) [89] is the first security system for WSNs, which was proposed by Perrig A on 2002. SPINS can provide data authentication, replay protection, and communication cost is low. Based on SPINS, extended security frameworks were proposed [90,91]. TinySec [92] is another lightweight link layer security architecture for WSN, which can provide authentication, integrity and confidentiality. Some research utilized [93] or extended [94] TinySec for WSNs. Beyond these two widely used security systems, there are also some other security systems, such as Minisec [95] and SHARP [96].

Although much security research mentioned above has been undertaken for UWSNs, almost all of them focused on a specific issue and have not been empirically verified. In [62], the existing secure communication protocols were discussed and compared carefully in detail.

As far as we can ascertain, there is still no practical, system-level security system for UWSNs. Hence, this will be a challenging topic and new trend for UWSNs.

## 5. Discussion and Future Research Topics

As discussed in previous sections, due to UWSNs’ particularities and constraints, mature technologies and systems cannot be directly utilized in them. Even though some security technologies have been investigated to secure UWANs, but almost of them are still in the theory stage, and empirical research is rare. Moreover, few proposals addressed several security issues systematically. UWSNs security research is still in its early stage, and moreover, several important issues have not been solved adequately.

In addition, network security is a complex cross-layer issue, and an optimal utilization of various security technologies among these layers is most important to minimize extra resource consumption, especially for severe resource-constrained UWSNs.

As discussed above, UWSNs are deployed underwater, and it is difficult to protect each underwater node and detect compromised nodes. The security schemes are pre-installed in the sensor nodes. If the node is compromised and rejected in the network, it will cause more severe damage. In this case, it is necessary to reconfigure security systems periodically, while the design and realization of such reconfiguring systems is also a challenge and important issue.

## 6. Conclusions

In this article, the challenges, threats and security issues in UWSNs are reviewed. Firstly, a brief introduction about UWSNs is presented. In the second part, the peculiarities and constraints of UWSNs and underwater environments are analyzed. Due to these particularities and constraints, UWSNs are vulnerable to a wide range of security threats and malicious attacks, which were discussed in Section 3. These attacks can severely disturb the communication and cooperation of the network. To avoid these attacks and guarantee the formal function of the network, the security requirements of UWSNs are introduced in Section 4. Furthermore, some specific security technologies and security schemes are discussed and analyzed. In Section 5, we discussed the existing security technologies and security systems, and introduced the new trend and challenging topics for UWSNs.

As discussed in this article, it is not easy to secure UWANs due to their peculiarities and constraints as well as the high cost of network deployment and maintenance. On the other hand, the particularities of UWANs also impose challenges, and should be leveraged in the design of security schemes. Moreover, applications may have different requirements in terms of security, and excessive security schemes will be a heavy burden in terms of energy consumption. Hence, how to take into account these features in security scheme design is also an important issue in future research.

## Figures and Tables

**Table 1:** Communication bandwidth.

	Long Range	Medium Range	Short Range
Communication Range	20–2000 km	1–10 km	<1 km
Working Environment	Deep Water	Shallow Water	Deep Water
Available Bandwidth	500 Hz–10 kHz	10–100 kHz	100–500 kHz
Data Rate	<10 kb/s	<50 kb/s	>100 kb/s

**Table 2:** Acoustic wave loss model.

	Signal Source	Working Environment	Spreading Loss
Spherical Wave Loss Model	point source	deep water	proportional to the square of the distance
Cylindrical Wave Loss Model	long line source	shallow water	proportional to the distance

**Table 3:** Spreading loss model for different communications range.

	Long-Distance	Medium-Distance	Short-Distance
Spreading Loss Model	cylindrical wave	cylindrical wave and spherical wave	spherical wave
Spreading Loss Factor	*d* = 1	*d* = 1.5	*d* = 2
